# An optimal degree of physical and chemical heterogeneity for the origin of life?

**DOI:** 10.1098/rstb.2011.0140

**Published:** 2011-10-27

**Authors:** Jack W. Szostak

**Affiliations:** Howard Hughes Medical Institute, and Department of Molecular Biology, and Center for Computational and Integrative Biology, Massachusetts General Hospital, Boston, MA 02114, USA

**Keywords:** origin of life, protocell, vesicle, fatty acids, genetic polymer

## Abstract

The accumulation of pure, concentrated chemical building blocks, from which the essential components of protocells could be assembled, has long been viewed as a necessary, but extremely difficult step on the pathway to the origin of life. However, recent experiments have shown that moderately increasing the complexity of a set of chemical inputs can in some cases lead to a dramatic simplification of the resulting reaction products. Similarly, model protocell membranes composed of certain mixtures of amphiphilic molecules have superior physical properties than membranes composed of single amphiphiles. Moreover, membrane self-assembly under simple and natural conditions gives rise to heterogeneous mixtures of large multi-lamellar vesicles, which are predisposed to a robust pathway of growth and division that simpler and more homogeneous small unilamellar vesicles cannot undergo. Might a similar relaxation of the constraints on building block purity and homogeneity actually facilitate the difficult process of nucleic acid replication? Several arguments suggest that mixtures of monomers and short oligonucleotides may enable the chemical copying of polynucleotides of sufficient length and sequence complexity to allow for the emergence of the first nucleic acid catalysts. The question of the origin of life may become less daunting once the constraints of overly well-defined laboratory experiments are appropriately relaxed.

## 1. Introduction

Experimental studies of prebiotic chemistry based on very simple initial conditions are often used to simulate chemical or physical processes that may have operated on the early Earth. In general, the outcomes of such experiments range from extremely heterogeneous mixtures of products to intractable tars. Classical examples are the alkaline formose reaction, which typically yields dozens of sugars, and the Miller–Urey spark discharge experiments, which yield hundreds of diverse compounds including dozens of amino acids (reviewed in [1]). Such studies are valuable in that they reveal potential means for the synthesis of biological building blocks, but they raise the problem of how biology could emerge from such complex mixtures. A similar puzzle arises in the case of the extreme heterogeneity of the organic chemical inventories of the carbonaceous meteorites. This complexity suggests that geochemical processes, such as might be found on a planet, might be needed to lead to simpler and more productive product mixtures.

Other experimental investigations lie at the opposite extreme, and are based on highly constrained and artificial model systems in which the transformation of a small number of pure, concentrated substrates into a restricted range of products is characterized in great detail. The classical example of such studies is the template-directed polymerization of activated nucleotides to yield partial complementary strands [2]. Such studies are necessary to obtain a detailed understanding of the chemistry underlying key reactions, but they suffer from the criticism of being prebiotically unrealistic and therefore a poor guide to processes that may have actually contributed to the emergence of biology from chemistry. In any case, such experiments have so far failed to guide us to a robust mechanism for the chemical replication of any informational polymer.

Several recent experiments suggest that there exists a middle ground between the unconstrained and the oversimplified, in which more realistic physical and chemical conditions lead to restricted sets of products that are potentially more useful for the construction of biology. This kind of controlled complexity is sometimes referred to as ‘systems chemistry’, and it has been invaluable in advancing studies of the prebiotic chemistry of nucleotide synthesis. In this approach, certain compounds may play multiple roles, e.g. as buffer, catalyst and reactant, exemplified by the role of phosphate in the pyrimidine synthesis pathway described by the Sutherland laboratory [3]. In this paper, I will review studies of membrane vesicle growth and division, primarily from my laboratory, and show how the relaxation of constraints that were designed to yield simple, homogeneous and reproducible vesicle populations eventually led to the identification of a simple and robust pathway for growth and division that operates on the heterogeneous vesicle populations that are formed by simple, natural processes. I will then consider the topic of nucleic acid template-directed replication from the same perspective.

## 2. Vesicle replication

As early as the 1970s, it was recognized that phospholipids were probably not the most appropriate building blocks for the membranes of early cells, largely because of the low permeability of phospholipid membranes to polar and charged small molecules. This property would severely limit the ability of primitive cells to take up nutrients synthesized in the external environment through abiotic chemical processes. Attention was, therefore, directed to the fatty acids as potential building blocks for prebiotic membranes. Fatty acids are components of phospholipids, and as simpler molecules are more likely to be available through abiotic syntheses. Early work of Gebicki & Hicks [4] and Hargreaves & Deamer [5] showed that fatty acids could self-assemble into bilayer membranes under the appropriate conditions, the most important of which are concentration and pH. Because fatty acids are single chain amphiphiles, the critical concentration necessary for stability of the membrane phase is much higher than for phospholipids of comparable chain length. In addition, the carboxylate group must be roughly half-protonated and half ionized to lead to a stable bilayer phase, presumably through the formation of hydrogen-bonded pseudo-dimers [6]. Once it was established that simple fatty acids could self-assemble into membrane vesicles, it became clear that such vesicles were a good model for the membranes of early protocells. Interestingly, mixtures of amphiphiles including fatty acids, fatty alcohols, fatty acid glycerol esters and even polyaromatic hydrocarbons generate membranes with superior thermostability and permeability compared with membranes made from individual, pure fatty acids [7–9]. Since fatty acid vesicles self-assemble quite easily, the question then became how could such vesicles grow and divide in the absence of complex and highly evolved cellular machinery?

In a speculative and theoretical article, Dave Bartel, Pier Luigi Luisi and I proposed that the spontaneous replication of primitive cell membranes was an essential aspect of the origin of life, comparable in importance to the replication of the genetic polymers responsible for inheritance [10]. Our reason for making this proposal was the idea that some form of spatial localization is essential for the emergence of Darwinian evolutionary processes, because localization allows for the functional activity of a genetic molecule to provide a selective advantage for itself. Membranes provide a simple means for the spatial localization of nucleic acids, and are of course universally used as cell boundaries by all modern living cells. Because the first protocells would, by definition, have lacked complex and highly evolved biochemical machinery, the growth and division of protocell membranes must have been driven by environmentally controlled physical and chemical forces. The nature of those forces and of the corresponding processes, therefore, became the focus of our attention.

Our initial experimental efforts in this direction focused on the process of vesicle growth following the addition of fatty acids in the form of alkaline micelles [11]. This process had been studied previously by the Luisi laboratory, primarily using dynamic light scattering (DLS) and cryo-electron microscopy to monitor vesicle growth [12,13]. Because we wished to develop a real-time method for the analysis of vesicle growth, we used a fluorescence assay for membrane growth based on the dilution of donor and acceptor dyes, and therefore a decreasing Förster resonance energy transfer efficiency, in a growing membrane. This assay was originally developed to follow the fusion of phospholipid vesicles, but worked very well in our hands to monitor the growth of fatty acid vesicles following the addition of ‘food’ molecules. In order to confirm these indirect growth measurements by independent methods, such as DLS and flow-field fractionation (FFF), we used small, unilamellar vesicles generated by extrusion. Progressive extrusion through smaller pores down to 100 nm in diameter was known to lead to a relatively homogeneous population of vesicles, almost all of which were composed of a single bilayer membrane [11]. Thus, we were driven by technical considerations towards a highly constrained and simplified experimental model system. Nevertheless, these experiments showed that slow addition of alkaline micelles to a buffered suspension of vesicles allowed for highly efficient transfer of fatty acids from the micelles into the preformed vesicle membranes.

**Figure 1.**
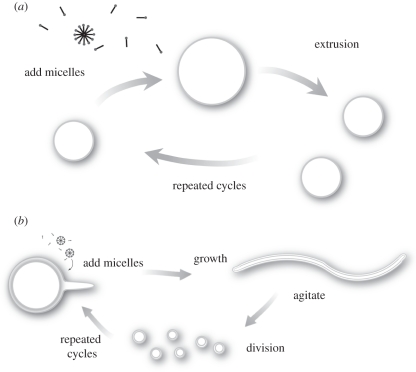
Growth and division of model protocell membranes. (*a*) Constrained, simplified model involving small monodisperse, unilamellar vesicles that grow following the addition of fatty acid micelles, and which divide during extrusion through small pores in a polycarbonate filter. (*b*) More plausible model in which heterogeneous large, multilamellar vesicles grow into filamentous vesicles following micelle addition, followed by division triggered by mild shear forces ((*b*) is adapted from fig. 1*c* of Zhu & Szostak [14] with permission).

While vesicle growth therefore seemed relatively simple and efficient, the issue of a physically driven means of division was more problematic, as was the development of a simple assay by which to monitor the efficiency of division. The process of extrusion through small pores is widely used as a standard method of producing small unilamellar and roughly monodisperse vesicles. Ultimately, we decided to adapt this same process as an expedient means of forcing vesicle division to occur. We found that when 100 nm vesicles had been grown to about 140 nm in diameter, thus doubling their surface area, they could be forced to divide back to an average size of 100 nm by a single passage through a filter of 100 nm pores [12]. This simple physical process thus allowed for a cycle of growth and division, which could be repeated indefinitely (figure 1*a*). This cycle is analogous to the cell division cycle in the important sense that both vesicle contents and vesicle membrane were distributed into daughter vesicles at each division. These experiments provided a proof of principle that vesicle growth and division could be driven by external physical forces, as opposed to being internally driven by cellular machinery as in biological cells.

Despite its simplicity, the above system was certainly deficient in terms of prebiotic plausibility. As noted above, the experiments were done with a highly artificial population of vesicles made by extrusion specifically to generate a relatively uniform size distribution, which was essential in order for us to detect growth in the population size distribution by methods such as light scattering. This contrasts with the extremely heterogeneous size distribution observed for vesicles formed by the hydration of dried lipid films, or by spontaneous assembly following the neutralization of alkaline micelles. Vesicle growth by the addition of alkaline micelles to neutral vesicles requires a somewhat elaborate, if not totally implausible geochemical scenario. More significantly, the process of division by extrusion has no plausible natural equivalent. Extrusion requires fluid flow under pressure through small pores, while flow through porous rock occurs almost entirely through larger fractures. Division of vesicles in a pond or lake could not occur through extrusion. We therefore continued to search for simpler and more plausible processes for both growth and division.

We were first able to demonstrate an alternative growth pathway that operates by competition between vesicles when we studied a slightly more complex and integrated system [15]. This discovery emerged from a consideration of the properties of model protocells consisting of a self-replicating nucleic acid genome encapsulated within self-replicating vesicles. Preliminary calculations suggested that a high concentration of encapsulated nucleic acid would lead, through its associated counter-ions, to a significant internal osmotic pressure, which would lead to vesicle swelling and thus membrane tension. We thought that this tension might provide a thermodynamic driving force for the preferential integration of new fatty acids into the tensed membrane, or even a re-distribution of lipids between tensed and relaxed membranes. Such a redistribution could occur because fatty acids are only loosely anchored within their membrane, with rapid dissociation and re-integration occurring on the sub-second time scale [16].

Our speculations were confirmed by experiments with osmotically swollen vesicles and isotonic relaxed vesicles. Both populations were stable separately (in that the vesicle sizes did not change with time), but as soon as they were mixed, the swollen vesicles started to grow while the relaxed vesicles began to shrink. Growth was observed with a variety of fatty acid-based vesicles, but not with phospholipid vesicles, which lack the dynamic exchange properties of fatty acid vesicles and are thus kinetically trapped in the swollen state. We observed osmotic pressure-driven growth using simple osmolytes such as sucrose, but also with mononucleotides and then with RNA. This was very exciting because of the implication that an encapsulated RNA replicase could drive protocell growth purely through the process of nucleic acid replication: the increased internal osmotic pressure resulting from the accumulation of trapped nucleic acids (as opposed to equilibrating monomers) would lead to membrane tension, and thus the ability to absorb fatty acids from the surrounding empty vesicles. Furthermore, in a population of vesicles containing replicating RNAs, any mutation that led to faster or more efficient replication would lead to faster cell growth as a whole, and thus a shorter cell cycle assuming that there was some stochastic or threshold-triggered process of cell division. Thus, the coupling of nucleic acid replication to membrane growth through osmotic pressure is a simple physical mechanism that could lead to the initiation of competition between protocells and thus the beginnings of Darwinian evolution. However, the nature of the physical processes that could lead to division in such a scenario remained obscure. This problem is made more difficult by the fact that osmotically swollen vesicles grow as spheres, for which a large energy input is needed to deform the spherical particle and drive division. Furthermore, geometrical constraints imply that a significant fraction of the internal volume must be lost to the environment during such a division process, since the volume of two spheres is less than the volume of one sphere with the same total surface area. The ideal pathway for division would therefore involve growth into a shape with a sub-spherical volume.

The solution to the problem of a simple and prebiotically plausible means of cell division emerged from experiments with a significantly more heterogeneous and less constrained vesicle population. We wished to gain insight into the process of growth by microscopic observations, which required the use of much larger vesicles than the small 100 nm vesicles we had used previously. As noted above, preparations of micrometre-sized vesicles tend to be extremely heterogeneous, in both size and lamellarity. This size heterogeneity is problematic if one wishes to observe a change in size distribution through microscopic (or other) observations. We therefore began by developing a simple means of preparing large fluorescently labelled vesicles that were relatively monodisperse, by using extrusion to remove vesicles above an upper size limit, followed by dialysis against large pore membranes to remove vesicles below a lower size limit [17]. The remaining vesicles, while similar in size, are still heterogeneous in terms of the number of bilayer membranes that comprise the outer vesicle boundary, as well as in the presence of smaller vesicles trapped within the larger vesicle. Nevertheless, we proceeded to examine the growth of such vesicles by simply adding alkaline micelles to buffered vesicles, and observing the process of growth in the microscope [14]. We expected to observe slow, uniform growth of the spherical vesicles to larger spherical or elongated vesicles, but the actual results were much more surprising. Bizarrely, the initially spherical vesicles sprouted short, thin tails in the first few minutes, and over the next half-an-hour these filamentous structures grew in length and diameter while the initial vesicle shrank and its components became distributed throughout the length of the long, branched filamentous vesicle. It was immediately apparent that this unanticipated growth pathway provided the long-sought solution to the problem of protocell division; because the filamentous vesicles were extremely fragile, even very mild shear forces caused by air currents or pressure-driven fluid disturbances were sufficient to trigger division of the filamentous vesicles into multiple smaller spherical daughter vesicles (figure 1*b*). Corresponding prebiotic scenarios of protocell division, e.g. triggered by wind-driven waves on the surface of a pond, are quite satisfying, especially by comparison with extrusion-driven division.

The unexpected nature of the spherical to filamentous vesicle transition led us to conduct further experiments aimed at understanding the mechanism underlying this phenomenon, as well as clarifying the conditions under which this pathway might operate. The two most important aspects are vesicle lamellarity and osmotic constraints. Careful efforts to prepare unilamellar, large vesicles showed that such vesicles do not grow through the same pathway as multilamellar vesicles. In particular, there is no initial phase in which a small thin filament protrudes from a unilamellar spherical parental vesicle; instead, there is a gradual transition to a more elongated, cylindrical appearance. Such vesicles are also quite fragile, but shear forces tend to disrupt these vesicles, resulting in loss of vesicle contents to the external environment. Ultimately, the torn membrane fragments reassemble into closed vesicles, and remarkably these are usually multilamellar. Thus, the multilamellar state appears to be in some sense a ground state, although access to this state is probably kinetically controlled. The second important constraint is osmotic. The formation of a filamentous vesicle requires that volume increase be osmotically constrained to be slower than surface area increase, since this results in transformation from a spherical to a low-volume morphology. Vesicle growth in the presence of a highly permeable buffer, ammonium acetate, did not result in filamentous growth. Instead, the outermost bilayer of a multilamellar vesicle was observed to balloon outwards as it grew rapidly following micelle addition. It is possible, though not yet proved, that the slow volume increase observed in the presence of slowly permeable solutes allows the multiple bilayers to remain closely apposed during the later stages of growth, favouring the exchange of materials between bilayers and thus the concerted growth of all bilayer membranes. This would lead to the observed multilamellar state of the post-growth filamentous vesicle, as well as the conservation of the mutlilamellar state following shear-induced division.

Slow volume increase relative to surface area increase requires the presence of slowly permeable solutes in the environment. This is an additional increase in the chemical complexity of the protocell and its environment, but one that appears to be quite reasonable. Our initial experiments were carried out with bicine buffer, which permeates across fatty acid membranes very slowly, but is not a prebiotically reasonable solute. We therefore carried out experiments in which we used a variety of other solutes, including a mix of the most common amino acids from Miller–Urey type syntheses, or from carbonaceous chondrite meteorites, which are glycine, alanine, valine and aspartate. Vesicle growth experiments in this amino acid milieu as a buffer showed the characteristic filamentous growth pathway. Finally, we were able to observe this growth and division pathway using a variety of fatty acid vesicle compositions, including a model prebiotic mixture of amphiphiles consisting of decanoic acid, decanol and decanoic acid-monoglycerate. Thus, this pathway for protocell growth and division appears to be quite robust to a variety of prebiotic scenarios, with the main constraints being that growth is initiated by the episodic addition of fatty acid micelles to preformed vesicles, in an environment containing slowly permeable solutes such as amino acids. Finally, the fact that division is fundamentally coupled to growth (since filaments can divide but spheres cannot) addresses one of the commonly raised questions about the coordination of growth and division in primitive cells lacking internal regulatory machinery.

We have recently observed another example of a modest increase in chemical complexity leading to a great simplification of the process of prebiotic vesicle growth and division. These experiments stemmed from an effort to identify the selective pressures responsible for the transition from primitive fatty acid-based membranes to modern phospholipid-based membranes [18]. Because phospholipid membranes are relatively impermeable to polar solutes, and lack the dynamic exchange properties that allow for vesicle growth, the earliest stages of this transition must have involved membranes that were predominantly composed of fatty acids (and related single chain amphiphiles), with only a small admixture of phospholipids. Such a membrane composition would be expected if the phospholipid was generated internally by a ribozyme with phospholipid synthase activity, since only a small amount of phospholipid would be generated initially owing to the low activity of nascent ribozymes. Ribozyme-catalysed phospholipid synthesis also requires the assumption that the necessary activated fatty acid substrates could be generated through prebiotic chemistry in the protocell environment. It would be very interesting if chemistry similar to that leading to the synthesis of activated nucleotides could also lead to the synthesis of activated fatty acids (e.g. thioesters or phospho-carboxy anhydrides).

For phospholipid synthesis activity to spread throughout the population, and for this activity to increase, it must have conferred some selective advantage. But what selective advantage would accrue from the synthesis of a relatively small amount of phospholipid by internal catalysis? Remarkably, vesicles whose membranes contain a small fraction of phospholipid are able to grow by absorbing fatty acids from neighbouring vesicles that do not contain phospholipids (or contain less phospholipid) [18]. The growth is rapid, and leads to the same filamentous morphology previously observed following fatty acid micelle addition. The resulting filamentous vesicles also divide when disturbed by mild shear forces. Thus, the ability to synthesize even low levels of phospholipid, from an internal catalyst, would lead to a strong selective advantage by allowing such vesicles to grow by absorbing fatty acids from neighbouring vesicles. Phospholipid-driven growth is a competitive growth pathway, which can occur in a steady-state environment, i.e. neither continuous nor sporadic input of additional fatty acids is required. As a result, the geophysical context in which protocell replication and Darwinian evolution could emerge is much simpler. All that is required is a pond or lake, acting as a reservoir for the accumulation of sufficient fatty acids, and the fatty acid derivatives that are the substrates for phospholipid synthesis, along with nucleic acid precursors, to self-assemble into vesicles containing replicating nucleic acids.

It is striking that a simple, robust pathway for vesicle growth and division has emerged in a few steps, largely involving the relaxation of initial artificial laboratory constraints. By going from small, homogeneous and well-characterized unilamellar vesicles to much more heterogeneous populations of large multilamellar vesicles, new and previously unexpected pathways for growth and division have emerged, which greatly simplify this aspect of the emergence of the first cellular life forms.

## 3. Nucleic acid replication

The spontaneous emergence of ribozymes with phospholipid synthase activity would initiate competitive protocell growth and division, leading to the rapid takeover of the population by vesicles containing this heritable ribozyme activity. Clearly, the most difficult and least understood aspect of this scenario is the process of nucleic acid replication within the replicating vesicles. Given that the relaxation of artificial constraints resolved the problem of protocell membrane growth and division, it is reasonable to ask whether analogous changes might prove beneficial in tackling the apparently more difficult problem of nucleic acid replication (or more generally, the replication of the protocellular genetic material, whatever its nature). Efforts to find a purely chemical, i.e. non-enzymatic, means of driving the replication of genetic polymers were initiated and vigorously pursued by Orgel, his students and colleagues beginning in the 1970s [3]. Partial success was indeed achieved, in that the template-directed copying of RNA templates could be observed in the presence of appropriately activated nucleotides. However, a complete cycle of replication remained elusive, and detailed studies uncovered a series of seemingly intractable problems. The copying of AU-rich templates was extremely inefficient, and product oligonucleotides contained a mixture of 2′–5′ and 3′–5′ phosphodiester linkages, even when metal ions and leaving groups that favoured 3′-linkages were used. Even partial copying of RNA templates required very high concentrations of activated and highly purified nucleotides (typically 0.1 M), in the presence of corresponding high Mg^2+^ concentrations (also typically 0.1 M). Chain termination following the incorporation of nucleotides of the wrong enantiomer implied that chemical replication was dependent on pools of pure, concentrated nucleotide substrates [19]. This was parodied by Joyce & Orgel [2] as ‘The Molecular Biologist's Dream’, a chemically unrealistic scenario for the emergence of prebiotic replication, and used to support the concept of a simpler progenitor to RNA. More recently, however, the prebiotic synthesis of RNA has begun to seem more plausible, largely owing to the experiments of Sutherland and co-workers [3]. These experiments embody the concept of systems chemistry, and show how the careful and selective relaxation of constraints on reaction conditions can favourably influence the outcome. For example, the reaction of glycolaldehyde and cyanamide alone yields a heterogeneous mixture of products, but in the presence of phosphate yields almost pure 2-aminooxazole as a product. Remarkably this product can be further purified by sublimation, and used as the input to the next step in nucleotide synthesis. Similar geophysical and geochemical processes may have played essential roles at many stages in the origin of life, acting to constrain the relevant chemistry so that the necessary mixtures of building blocks at each stage were neither too simple nor too complex.

**Figure 2.**
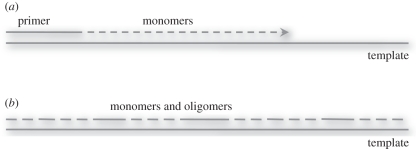
Copying of genetic polymers. (*a*) Constrained, simplified model in which a template is copied by the extension of a primer, one nucleotide at a time. (*b*) Less constrained, more plausible model involving the nucleation of copying at multiple sites, followed by gap filling with mono- and oligo-nucleotides.

Are there ways in which the artificially simplified conditions we use to study template-directed replication might be modified, so as to provide a higher yield of desired full length, high-fidelity products? First, I will explain the rationale behind our highly constrained (i.e. prebiotically unrealistic) model systems, and then I will discuss possible ways in which these experiments could become gradually more realistic. Our current experiments on nucleic acid replication are focused on a series of model polymers, the phosphoramidate nucleic acids, which form from the polymerization of amino-sugar nucleotides [20,21]. These monomers and the corresponding polymers may or may not be of prebiotic relevance—there have been few if any studies of potentially prebiotic pathways for their synthesis. However, they do provide a very convenient model for the study of the underlying principles of chemical replication. This advantage stems in a large part from the greater nucleophilicity of the amino group (relative to the normal sugar hydroxyl), which imparts a significantly enhanced reaction rate during template-copying experiments. As in past work, much of our exploration of these systems has made use of simple, analytically tractable primer-extension experiments, using highly purified monomer substrates (figure 2*a*). Such experiments are ideal for the initial characterization of a novel nucleic acid replication system. However, some problems, such as difficulty in copying A- and U-rich sequences, immediately became apparent in the 2′-amino, 2′,3′-dideoxyribo system [21]. The use of non-standard modified nucleotides designed to increase base-pairing strength [22] improved the rate of primer-extension, but may have simultaneously decreased the fidelity of the template-copying reaction. More subtle difficulties, such as an unexpected decrease in rate during attempts to copy long mixed sequence templates (compared with the rapid and efficient copying of short homopolymer templates) also became apparent.

In an effort to address these problems, we have begun to study the polymerization of short oligomeric substrates. Indeed many previous studies of template copying using oligonucleotide substrates have been carried out (figure 2*b*). The chemical ligation of oligodeoxynucleotides occurs with surprisingly high fidelity, and fidelity increases with increasing temperature [23]. Our own early studies suggested that the chemical ligation of oligoribonucleotides on RNA templates proceeded with excellent high 3′–5′ regiospecificity in the products [24]. The combination of good fidelity and good regiospecificity is a powerful argument in favour of a role for oligonucleotides in any primitive replication system. Oligonucleotide substrates would also speed up the copying process, since fewer chemical steps would be required, and copying could be nucleated at multiple points on the template. If oligonucleotides were the substrates for an early form of chemical replication, it seems likely that a very heterogeneous mix of lengths and sequences would have been used. Furthermore, replication relying only on oligomer substrates would seem inevitably to lead to copying intermediates separated by gaps of one, two or more nucleotides. The simplest solution may be to use a mixture of monomers and short oligomers, perhaps generated by a combination of template-directed and non-template-directed polymerization, as substrates for template copying. The experimental investigation of such copying reactions poses analytical challenges, especially if one abandons the use of convenient (but artificial) end-labelled primers. Fortunately, modern high-resolution mass spectrometry provides the ideal tool for analysing the products of template copying from such complex reaction mixtures. We are currently using this approach to investigate template-copying reactions with heterogeneous mixtures of substrates.

The use of heterogeneous substrate pools can also, in principle, be extended to experimental searches for an RNA replicase (or any other nucleic acid replicase). To date, most efforts to evolve a replicase have focused on the *in vitro* evolution of ribozyme polymerases that are closely analogous to modern protein RNA polymerase enzymes, in that they convert activated monomers to a full-length template copy. However, if we begin from a less constrained conception of chemical replication, in which the substrates are a heterogeneous mixture of monomers and short oligomers, possibly mixed with their cyclized side-products, 2′–5′ linked oligomers and a diversity of non-RNA species, then there are many ways that simple ribozymes could contribute to an enhanced replication rate and fidelity. For example, a ribozyme could act to complete the copying of a partially copied template, in which short gaps remain—such gap filling activities have already been reported [25]. Alternatively, an exonuclease activity could clean up the partial products of template copying. This might have been highly advantageous, since the low fidelity of chemical copying is likely to result in frequent mismatch generation, which greatly slows down subsequent chain elongation [26]. Excision of a terminal mismatch (or 2′–5′ linked nucleotide or a non-ribonucleotide) would allow chemical copying to proceed, and would greatly increase the accuracy of the fully replicated products. Similarly, cyclic-phosphodiesterase and endonuclease activities that would regenerate useful substrates from cyclic mono- and oligo-nucleotides would be highly advantageous. Through the evolution of a panel of such ribozymes, the process of genomic replication could gradually shift from a purely chemical process to one driven by an array of genomically encoded catalysts, in the process allowing for a gradual increase in the length and informational complexity of the primitive genome.

## 4. Conclusions

Initial studies of complex and poorly understood phenomena often make use of simplified laboratory models; however, as our understanding increases and our technical abilities improve, it becomes both possible and necessary to investigate increasingly realistic models of the phenomenon in question. Just such changes are occurring in diverse aspects of studies of the origin of life, such as the prebiotic chemistry of nucleotide synthesis, the growth and division of model protocell membranes and the replication of primitive genetic polymers. In the first two cases, moderate increases in initial chemical or physical complexity led to solutions to problems that previously seemed intractable, and in the third case, an analogous approach looks promising. The argument in favour of an appropriate degree of chemical complexity has been made very cogently by Sutherland and co-workers [3] for prebiotic nucleotide synthesis. In the case of protocell membranes, our efforts to prepare a well-defined, analytically tractable vesicle system initially prevented us from observing a prebiotically plausible pathway for protocell division. Only later, when we started to work with more natural vesicle preparations, were we able to discover such a pathway [14]. Most work on the chemical replication of genetic polymers has involved well defined and analytically tractable model systems. A shift from the use of pure, concentrated monomers to drive template-directed primer-extension, to the use of more complex mixtures of monomers and short oligomers in primer-free template copying may ultimately be required in order to achieve complete cycles of chemical replication. Scenarios involving moderate chemical and physical complexity are, in general, more geochemically sensible, and thus more prebiotically plausible than over-simplified laboratory models. It would, therefore, be very satisfying indeed if such scenarios turned out to be not only compatible with but also necessary for the key steps in the chemical origins of life.
